# Effect of Fluoride Varnish Containing CPP-ACP on Preventing Enamel Erosion

**DOI:** 10.1155/2017/1897825

**Published:** 2017-01-09

**Authors:** Sule Bayrak, Nuray Tuloglu, Huseyin Bicer, Emine Sen Tunc

**Affiliations:** ^1^Department of Pediatric Dentistry, Faculty of Dentistry, University of Eskisehir Osmangazi, Eskisehir, Turkey; ^2^Department of Pediatric Dentistry, Faculty of Dentistry, University of Ondokuz Mayıs, Samsun, Turkey

## Abstract

This study aimed to investigate the effect of a fluoride varnish with added casein phosphopeptide-amorphous calcium phosphate treatment on the prevention of enamel erosion, and it compared the results with those of other fluoride varnishes. Fifty enamel specimens obtained from bovine incisors were randomly divided into five groups (*n* = 10) based on the type of surface pretreatment used: intact enamel (Group 1); intact enamel+erosive cycles (Group 2); intact enamel+MI varnish+erosive cycles (Group 3); intact enamel+Clinpro White varnish+erosive cycles (Group 4); and intact enamel+Duraphat varnish+erosive cycles (Group 5). The specimens were subjected to erosive cycles for five days. The surface roughness was evaluated using atomic force microscopy. The results were statistically analyzed using one-way ANOVA and Tukey's tests. Group 1 had the smoothest surfaces. After the erosive cycles, the greatest surface roughness values were observed in Group 2, followed by Groups 5, 4, and 3, respectively. Statistically significant differences were observed among all groups (*p* < 0.05). The application of fluoride varnishes had some positive effects on preventing enamel erosion; however, the most effective agent was fluoride varnish with added casein phosphopeptide-amorphous calcium phosphate.

## 1. Introduction

Dental erosion is defined as an irreversible loss of dental hard tissues by the chemical dissolution process initiated by acids of nonbacterial origin or chelation when the surrounding aqueous phase is undersaturated with respect to tooth mineral [1, 2]. The etiology of dental erosion is multifactorial and includes chemical, biological, and behavioral factors [3]. To prevent the occurrence of dental erosion, resources for diet guidelines may be used, as well as the application of products that minimize demineralization and promote remineralization of the tooth structure [4].

Topical applications of highly concentrated fluorides, such as oral rinses, gels, or varnishes, have been considered to prevent the dissolution of enamel and increase the resistance of enamel against erosive attacks [5–9]. The effect of fluorides is mainly related to the formation of a calcium fluoride- (CaF_2_-) like layer precipitate on the enamel surface, which acts mainly as a mineral reservoir and which can partially behave as a physical barrier avoiding contact between the acid and the underlying enamel [5, 10, 11].

In addition to fluoride, products based on calcium and phosphate can be an alternative for preventing tooth enamel erosion [12–18]. Topically administered casein phosphopeptide-amorphous calcium phosphate (CPP-ACP) buffers free calcium and phosphate ion activity, maintaining a state of supersaturation with respect to tooth enamel that helps prevent demineralization and facilitates remineralization on dental caries or erosion [19].

Recently, more advanced fluoride varnishes with added CPP-ACP have been developed [19]. Numerous reports have been published on the protective effect of CPP-ACP and casein phosphopeptide-amorphous calcium fluoride phosphate (CPP-ACFP) paste/solution and the synergistic effect of CPP-ACP and fluoride on erosion [13–17, 20–25]. However, to our knowledge, no previous studies have evaluated the ability of fluoride varnish with added CPP-ACP to increase tooth enamel's resistance to erosion. Therefore, the present study investigated the effects of fluoride varnish with added CPP-ACP treatments on the prevention of enamel erosion, and it compared the results with those of other fluoride varnishes. Two null hypotheses tested were as follows: (1) fluoride varnish containing CPP-ACP does not increase the enamel's resistance to erosion and (2) there are no significant differences in the prevention of enamel erosion among different types of fluoride varnish treatments.

## 2. Materials and Methods

### 2.1. Materials

Three different fluoride varnishes were evaluated: MI Varnish (GC, Tokyo, Japan), Clinpro White Varnish (3M Espe, MN, USA), and Duraphat varnish (Colgate-Palmolive, NSW, Australia). Details of the varnishes are represented in Table 1.

### 2.2. Preparation of Enamel Specimens

The enamel specimens (4 mm × 4 mm × 3 mm) were prepared from freshly extracted bovine incisor teeth. Specimens with cracks, stains, or white spot lesions were excluded, and the selected teeth were stored in 0.1% thymol solution (pH = 7.0) at 4°C prior to the experiment.

Fifty included specimens were embedded in an acrylic resin cylinder with the enamel surface exposed. The specimens were ground under running water using a polishing machine with 320-, 600-, and 1200-grit silicon-carbide papers, removing about 200 *μ*m of the surface of the tooth [26]. Thereafter, the specimens were cleaned in an ultrasonic device (Isolab Laborgeräte GmbH, Wertheim, Germany) with deionized water for five minutes. The specimens were covered with two layers of acid-resistant nail varnish, leaving an exposed window of enamel, approximately 1 mm × 1 mm, in the center of each buccal surface.

Baseline root mean-square roughness (*R*_rms_) was measured for all the specimens before beginning the experiment, and it was observed that *R*_rms_ values of the specimens may be comparable.

### 2.3. Treatment Protocols and Erosive Cycles

The enamel specimens were randomly divided into five groups (*n* = 10) based on the type of enamel surface pretreatment used, as follows:

- Group 1: intact enamel (no treatment, negative control group).
- Group 2: intact enamel+erosive cycles applied (positive control group).
- Group 3: intact enamel+MI varnish+erosive cycles applied.
- Group 4: intact enamel+Clinpro White varnish+erosive cycles applied.
- Group 5: intact enamel+Duraphat varnish+erosive cycles applied.

Before the erosive cycles, the fluoride varnishes were applied in a thin layer using a microbrush, and the specimens were stored in artificial saliva at 25°C for six hours [10, 26, 27]. The varnishes were then carefully removed with acetone solution (1 : 1 water) and a plastic scaler in an effort to avoid touching the enamel surface [26]. Complete removal of the varnishes was checked microscopically (×40) (Nikon SMZ-1500, Osaka, Japan).

The specimens underwent erosive demineralization by immersion in 1.0% citric acid (pH 3.6, 10 mL/specimen) for 90 seconds, four times a day for five days under constant agitation (70 rpm) (Shaker, Isolab Laborgeräte GmbH, Wertheim, Germany) [18, 26, 27]. After each demineralization, the specimens were rinsed with deionized water (10 seconds) and transferred to artificial saliva (pH 6.8, 10 mL/specimen, unstirred, 25°C) for two hours [26]. After the final daily erosive treatment, the specimens were also stored in artificial saliva overnight. The citric acid was renewed at each erosive challenge, and the artificial saliva was replaced daily.

### 2.4. Evaluation of Surface Roughness

Surface roughness is commonly represented as *R*_rms_, which is given by the standard deviation of the height. After undergoing five daily erosive cycles, *R*_rms_ of the specimens was evaluated using atomic force microscopy (AFM) (MultiMode 8, Veeco Instruments Inc., Plainview, New York, USA), operating in tapping mode. A Reduced Temperature Electrode Supported Planar probe (Bruker Nano Inc., Camarillo, CA, USA) was used to avoid damaging the softened enamel. *R*_rms_ for each specimen was obtained using a 10 × 10 *μ*m^2^ film area with a range of 1 *μ*m in the *z*-direction and a resolution of 256 × 256 pixels.

### 2.5. Statistical Analysis

Statistical analysis was performed using SPSS for Windows, Version 12.0.1 (SPSS, Inc., Chicago, IL, USA). One-way ANOVA was used to identify significant differences (*p* < 0.05) in *R*_rms_ among the five groups.* Post hoc* comparisons were made using Tukey's test. The level of significance was set at *p* < 0.05.

## 3. Results

**Figure 1:**
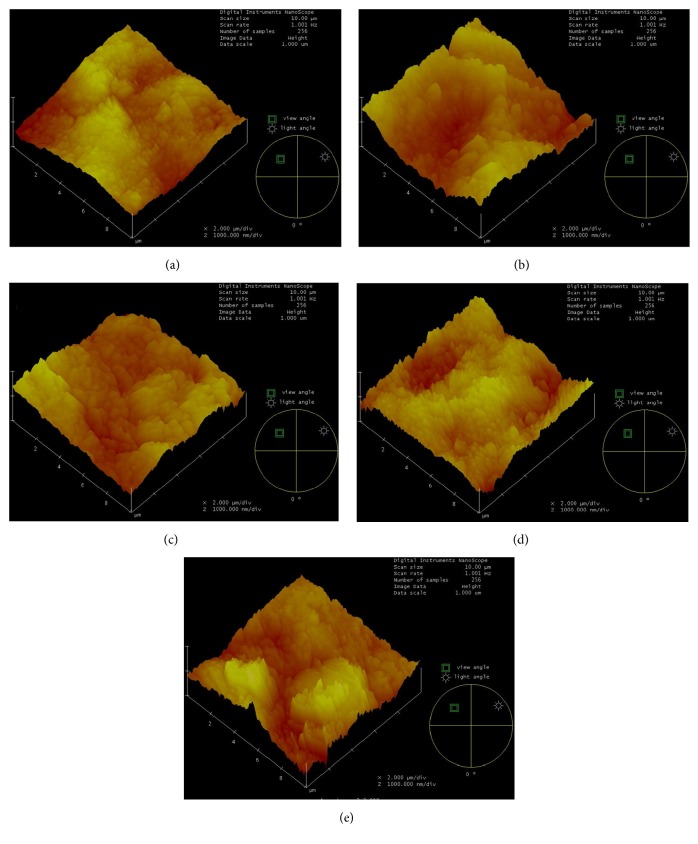
AFM images of erosion areas for all groups: (a) Group 1 (negative control); (b) Group 2 (positive control); (c) Group 3; (d) Group 4; (e) Group 5.

Means and standard deviations of the surface roughness values are given in Table 2, and the AFM images for all five groups are presented in Figure 1.

After the erosive cycles, surface roughness measurements ranged from 149.84 nm to 261.70 nm, with statistically significant differences among the groups (*p* < 0.05) (Table 2). Group 1 had the smoothest surfaces (Figure 1) with a mean surface roughness of 149.84 ± 15.84 nm. Comparing Group 1 with Group 2 (261.70 ± 25.99 nm), a statistically significant difference (*p* < 0.05) in *R*_rms_ values was found, with an increase in the surface roughness passing from the intact enamel to the enamel exposed to citric acid. Comparing *R*_rms_ values of Groups 3, 4, and 5 with Group 1, a statistically significant decrease (*p* < 0.05) in the surface roughness values was found, which suggests a remineralizing effect for all the varnishes.

Comparing *R*_rms_ values of all the varnish groups (Groups 3, 4, and 5) with Group 2, a statistical difference (*p* < 0.05) was found, suggesting that all the varnishes had a protective effect against enamel demineralization. This protective effect appeared to be more pronounced for the MI varnish, and this was confirmed with the morphological analysis of the AFM image (Figure 1).

Among the varnishes tested, the lowest mean surface roughness values were seen with Group 3 (171.18 ± 16.78 nm) followed by Group 4 (193.09 ± 8.38 nm) and Group 5 (214.57 ± 9.20 nm) (Table 2). Statistically significant differences were observed among all the varnish groups (*p* < 0.05) (Table 2).

## 4. Discussion

New materials are continually being introduced in dental practice. Not only do these materials require examination to confirm the properties they claim to possess, it is also important to propose modifications or new associations that can contribute to improving their performance. For years, the application of fluoride agents in various forms has been the most effective and frequently employed method used in the prevention of enamel erosion [5–9]. Recently, a fluoride varnish containing CPP-ACP became commercially available on the dental market [19, 28]; however, to the best of our knowledge, no published studies have reported on the product's effect on the erosion resistance of enamel. Consequently, this present study aimed to investigate the efficacy of fluoride varnish containing CPP-ACP on tooth enamel's resistance to erosion.

The present study was conducted using enamel specimens obtained from bovine teeth, which have also been used in previous studies [10, 12, 13, 23, 26, 29, 30]. Bovine teeth have long been used in experiments as a substitute for human teeth because of the similarities both types of teeth share with regard to chemical and physical properties, such as composition and hardness [31, 32]. Additionally, because the composition of bovine teeth has less variation than human teeth, the use of bovine teeth results in more standardized test conditions [31]. The chemical structure of bovine enamel and its reaction to erosive attack are also comparable to human enamel, and the size of bovine teeth helps ensure sufficient enamel surfaces. Moreover, it is advantageous to use bovine specimens because up to four-five specimens can be gained from a single bovine incisor [33].

Shellis et al. [27] recommended that specimens should be stored in moist conditions between studies or between cycles. In order to simulate clinical conditions and standardize the experimental conditions [34], the specimens were stored in artificial saliva at 25°C throughout the course of the experiment in this present study.

Many strategies have been used to prevent erosion in enamel, such as highly concentrated fluoride applications in the form of oral rinses, gels, or varnishes [5–9]. Fluoride varnishes may be more effective [35] because they provide long contact periods between the dental tissues and the fluoride agent, which results in high fluoride uptake and the formation of CaF_2_ deposits that act as fluoride reservoirs [5, 10, 11]. The protective effect of sodium fluoride against dental erosion has been shown in previous studies [5, 7, 8, 26]. In addition to fluoride, other minerals, such as calcium and phosphate, may be used to enhance the protective/strengthening benefits of fluoride to better address dental erosion [36]. The use of calcium and phosphate products together with fluoride has been reported to have a synergistic effect [37, 38]; therefore, a sodium fluoride varnish, a sodium fluoride varnish containing tricalcium phosphate, and a sodium fluoride varnish containing CPP-ACP were included among the treatment regimens that were tested.

A soft drink, fruit juice, or a simple acid solution is usually used to model the extrinsic agents. Citric acid is usually used to simulate a soft drink acid in dental erosion studies [11, 26, 29], and it can provide a strong erosive challenge under certain conditions [11]. A solution of 0.052 mol/L (1.0%) citric acid, pH 3.6, seems suitable, as its pH is within the range of 3.5–3.75 for orange juice and it has the same titratable acidity [27]. Moreover, the amount of time that the tooth surface is exposed to acid should be minimal, so that the surface change does not exceed the initial erosion, enabling it to be measured accurately [35]. In light of this information, the specimens used in this study were subjected to erosive cycles (4 × 90 s/day in 1.0% citric acid, intercalated with artificial saliva) for five days.

Many techniques, such as microradiography [5], quantitative light-induced fluorescence [39], surface hardness [8, 11, 12, 17, 18, 23, 29, 30], scanning electron microscopy [14], profilometry [6, 10, 13, 15, 20, 27, 30], and AFM [14, 21, 24], have been used to evaluate the effectiveness of agents on the prevention of enamel erosion. AFM is a nanoindentation technique that is capable of obtaining images with atomic resolution with minimal sample preparation [24, 39], and it has recently been used to study enamel erosion [14, 21, 24]. Specimen preparation is one of the main advantages of AFM over other techniques [40]. AFM can be used equally well on conducting and insulating surfaces, and it can be performed in ambient conditions, in air, or liquids, as well as in a vacuum [40]. Thus, fragile samples are not damaged by harsh sample preparation techniques, such as coating, dehydration, and exposure to a vacuum, and artefacts associated with such techniques are avoided [40]. Furthermore, the same sample can be imaged in AFM in real time [40]. A further advantage is that AFM is extremely accurate, so it is possible to obtain quantitative data [40]. Based on these advantages, this present study used AFM analysis to evaluate morphological changes on enamel after erosion.

Our results showed that fluoride varnish with CPP-ACP provided the most resistance against enamel erosion in comparison to the other fluoride varnishes. Fluoride varnish with CPP-ACP provides additional fluoride along with calcium and phosphate ions for remineralization. Previous studies have shown that CPP-ACP, CPP-ACFP, and CPP-ACP+fluoride containing agents can significantly increase hardness [12, 20, 23, 41] and decrease erosion [13, 14, 16, 21, 23, 41] of enamel softened by erosive substances. The actual mechanism of CPP-ACP on enamel erosion may involve the incorporation of nanocomplexes on the enamel surface. CPP-ACP nanocomplexes located on the enamel surface have been purported to buffer the activity of free calcium and phosphate ions, thereby maintaining a state of supersaturation with respect to tooth enamel, preventing enamel demineralization, and promoting remineralization [13, 21]. The treatment of CPP-ACP was also found to facilitate the formation of a crystal layer, filling the interprism, and partially covering the prisms, thus preventing acid attack [21]. In line with our results, Poggio et al. [21] and Ceci et al. [24] also demonstrated that treatment with CPP-ACP paste to prevent dental erosion reduced the surface roughness measurements, as shown by AFM. Therefore, the first null hypothesis of the present study was rejected.

This study compared the effects of different types of fluoride varnish treatments on the prevention of enamel erosion, and statistically significant differences were observed among all the varnish groups; therefore, the second null hypothesis was also rejected. The study's findings indicate that Clinpro White varnish offers greater protection against the challenge of enamel erosion than Duraphat varnish; however it reveals less protection than MI varnish. It may be explained by the high release of calcium and inorganic phosphate ions from MI varnish or by the low solubility of tricalcium phosphate from Clinpro White varnish [19]. The clinical efficiency of fluoride varnishes varies according to the amount of CaF_2_-like precipitate, which depends on the concentration of the applied fluorides and the amount of provided calcium ions. Fluoride varnishes are available commercially in many different forms and concentrations. One of the most widely used is 5% sodium fluoride. A fluoride varnish with added tricalcium phosphate is also currently available. Tricalcium phosphate is a hybrid material created with a milling technique that fuses beta tricalcium phosphate and sodium lauryl sulfate or fumaric acid [25]. This blending results in a functionalized calcium and a free phosphate, which is designed to increase fluoride retention in both enamel and dentine and facilitate remineralization [29]. When tricalcium phosphate comes into contact with the tooth surface and is moistened by saliva, the protective barrier breaks down, making calcium, phosphate, and fluoride ions available to the teeth [25]. In line with our findings, other studies [17, 25, 29, 42] have shown that tricalcium phosphate with fluoride can promote the protective effect in eroded enamel.

In the present study, all the fluoride varnish groups had lower mean surface roughness values than the positive control group. This is in accordance with the results of other studies [5–9, 35], which have shown that highly concentrated fluoride is able to protect enamel from erosion. The preventive effect demonstrated by the topical application of fluoride has also been attributed to its demonstrated ability to form calcium fluoride [5, 9–11]. While calcium fluoride leaches slowly and easily when challenged by acid, it does prevent the dissolution of minerals from enamel by providing a physical barrier on the enamel surface [43]. Our results are in accordance with other studies [7, 8, 10, 26, 30], which showed that sodium fluoride varnishes are effective in reducing the progression of tooth erosion and which found significantly lower values for surface roughness in all the fluoride groups when compared to a positive control group that was not treated with fluoride.

## 5. Conclusion

Within the limitations of the present study, the different fluoride varnishes that were tested were all found to have positive effects on the prevention of enamel erosion; however, the fluoride varnish containing CPP-ACP was the most effective in increasing the enamel's resistance to erosion. In order to confirm the data obtained from this* in vitro* study and investigate the effects of fluoride varnish containing CPP-ACP under clinical situations, clinical trials are required.

## Figures and Tables

**Table 1:** Varnishes used in this study.

Varnish	Content	Manufacturer	Lot number	Source
MI	30–50% polyvinyl acetate, 10–30% hydrogenated rosin, 20–30% ethanol, 1–8% sodium fluoride, 1–5% CPP-ACP, 1–5% silicon dioxide	GC, Tokyo, Japan	141009A	MSDS
Clinpro White	30–75% pentaerythritol glycerol ester of colophony resin, 10–15% n-hexane, 1–15% ethyl alcohol, 1–5% sodium fluoride, 1–5% flavour enhancer, 1–5% thickener, 1–5% food grade flavour, <5% modified tricalcium phosphate	3M Espe, MN, USA	N545905	MSDS
Duraphat	10–<40% colophonium, 10–<30% ethanol, <5% sodium fluoride, <1% saccharin, <1% isoamyl acetate, other ingredients	Colgate-Palmolive, NSW, Australia	BB2LX	MSDS

**Table 2:** Mean surface roughness values (*R*_rms_) ± standard deviation.

Groups	Mean ± standard deviation (nm)
Group 1 (negative control)	149.84 ± 15.84^a^
Group 2 (positive control)	261.70 ± 25.99^e^
Group 3	171.18 ± 16.78^b^
Group 4	193.09 ± 8.38^c^
Group 5	214.57 ± 9.20^d^

Different superscript letters indicate statistically significant differences (one-way ANOVA; Tukey's test; *p* < 0.05; *n* = 10).
